# Physician Attitudes toward Adopting Genome-Guided Prescribing through Clinical Decision Support

**DOI:** 10.3390/jpm4010035

**Published:** 2014-02-27

**Authors:** Casey Lynnette Overby, Angelika Ludtke Erwin, Noura S. Abul-Husn, Stephen B. Ellis, Stuart A. Scott, Aniwaa Owusu Obeng, Joseph L. Kannry, George Hripcsak, Erwin P. Bottinger, Omri Gottesman

**Affiliations:** 1 Program for Personalized and Genomic Medicine and Department of Medicine, University of Maryland School of Medicine, Baltimore, MD 21201, USA; E-Mail: coverby@medicine.umaryland.edu; 2 Center for Health-Related Informatics and Bioimaging, University of Maryland, Baltimore, MD 21201, USA; 3 Department of Medicine, Icahn School of Medicine at Mount Sinai, New York, NY 10029, USA; E-Mails: angelika.ludtke@mssm.edu (A.L.E.); noura.abul-husn@mssm.edu (N.S.A.-H.); aniwaa.owusu-obeng@mssm.edu (A.O.O.); 4 Department of Genetics and Genomic Sciences, Icahn School of Medicine at Mount Sinai, New York, NY 10029, USA; E-Mail: stuart.scott@mssm.edu; 5 The Charles Bronfman Institute for Personalized Medicine, Icahn School of Medicine at Mount Sinai, New York, NY 10029, USA; E-Mails: steve.ellis@mountsinai.org (S.B.E.); erwin.bottinger@mssm.edu (E.P.B.); 6 Division of General Internal Medicine, Icahn School of Medicine at Mount Sinai, New York, NY 10029, USA; E-Mail: joseph.kannry@mountsinai.org; 7 Department of Pharmacy, The Mount Sinai Hospital, New York, NY 10029, USA; 8 Department of Biomedical Informatics, Columbia University, New York, NY 10032, USA; E-Mail: hripcsak@columbia.edu; 9 Division of Nephrology, Icahn School of Medicine at Mount Sinai, New York, NY 10029, USA; 10 Department of Pharmacology and Systems Therapeutics, Icahn School of Medicine at Mount Sinai, New York, NY 10029, USA

**Keywords:** clinical decision support, genomic medicine, clinician perceptions

## Abstract

This study assessed physician attitudes toward adopting genome-guided prescribing through clinical decision support (CDS), prior to enlisting in the Clinical Implementation of Personalized Medicine through Electronic Health Records and Genomics pilot pharmacogenomics project (CLIPMERGE PGx). We developed a survey instrument that includes the Evidence Based Practice Attitude Scale, adapted to measure attitudes toward adopting genome-informed interventions (EBPAS-GII). The survey also includes items to measure physicians’ characteristics (awareness, experience, and perceived usefulness), attitudes about personal genome testing (PGT) services, and comfort using technology. We surveyed 101 General Internal Medicine physicians from the Icahn School of Medicine at Mount Sinai (ISMMS). The majority were residency program trainees (~88%). Prior to enlisting into CLIPMERGE PGx, most physicians were aware of and had used decision support aids. Few physicians, however, were aware of and had used genome-guided prescribing. The majority of physicians viewed decision support aids and genotype data as being useful for making prescribing decisions. Most physicians had not heard of, but were willing to use, PGT services and felt comfortable interpreting PGT results. Most physicians were comfortable with technology. Physicians who perceived genotype data to be useful in making prescribing decisions, had more positive attitudes toward adopting genome-guided prescribing through CDS. Our findings suggest that internal medicine physicians have a deficit in their familiarity and comfort interpreting and using genomic information. This has reinforced the importance of gathering feedback and guidance from our enrolled physicians when designing genome-guided CDS and the importance of prioritizing genomic medicine education at our institutions.

## 1. Introduction

Though a clear role has been established for pharmacogenomics in the efficacy and toxicity of numerous drugs, the implementation of pharmacogenomic tests in clinical practice has not kept pace with the emerging knowledge base [1,2]. There are numerous potential explanations for this lag, including an insufficient knowledge of and experience with pharmacogenomic testing [3,4,5]. The risk of pharmacogenomic interventions is generally low, and some suggest that pharmacogenomic testing need only reach evidence levels of non-inferiority compared with current prescribing practices to merit use [6]; however, others argue that additional evidence is still needed prior to implementation [7]. Additionally, though genetics has long played a part in undergraduate and postgraduate medical education, this has largely been limited to well-understood genetic defects that cause Mendelian disease. Genomics is a relatively new field and has rapidly transitioned from research to potential clinical implementation. This has left most of the current provider workforce ill prepared. As a result, studies surveying the knowledge, skills and confidence of the clinical workforce in implementing genomic medicine have revealed a deficit that must be addressed [8]. This deficit is particularly concerning in the context of physicians who are in their early postgraduate years. These physicians will likely encounter a significant and rapid increase in genome-related, clinically relevant information over the course of their careers, and without any formal training in the interpretation and use of this information could be left adrift.

The Clinical Implementation of Personalized Medicine through Electronic Health Records and Genomics pilot pharmacogenomics project (CLIPMERGE PGx) in progress at the Icahn School of Medicine at Mount Sinai (ISMMS) [9] focuses on presenting genetic variants with established clinical significance (e.g., gene/drug pairs with Clinical Pharmacogenetics Implementation Consortium (CPIC) guidelines [10]) to internal medicine physicians and trainees through patient electronic health records. Through this project, a novel clinical decision support (CDS) engine for delivering evidence in a way that integrates with physician work processes was developed [9].

A few studies have reported attitudes toward electronic health record (EHR) adoption [11,12], although more relevant are studies assessing attitudes toward prescribing CDS adoption. In one such study [13] researchers utilized the Information Technology Adoption in Primary Care Practice (ITAM) instrument [14,15,16] to investigate the influence of attitudes on the adoption of an e-prescribing system that provided dosing guidance, duplicate therapy checks, and weight-based pediatric dosing guidance. ITAM applies attitudes (e.g., perceived ease of use, perceived usefulness) to the use of computers in medical care. In contrast to these studies, given our interest in the adoption of new knowledge that may be unfamiliar to physicians, we designed a survey instrument that adapts the Evidence Based Practice Attitude Scale (EBPAS) [17,18] to measure attitudes toward adopting evidence-based practices. As such, our study assessed physician attitudes toward adopting genome-guided prescribing through CDS prior to enlisting in CLIPMERGE PGx. Another study investigating the influence of provider attitudes on the uptake of prescribing CDS deployed in a primary care setting is in progress.

## 2. Experimental

### 2.1. Setting

The CLIPMERGE PGx study was approved by Mount Sinai Institutional Review Board (IRB Approval ID GCO 12-0931). We recruited from the entire population of the Mount Sinai Medical Center’s Internal Medicine physicians, including residents in post-graduate year (PGY) 1 to 4, fellows, and attendings, who provide patient care at the Internal Medicine Associates (IMA) primary care facility. Before enlisting as a CLIPMERGE study participant, physicians were required to attend a one-hour teaching session outlining the study and CDS content.

### 2.2. Survey Design and Development

As a preliminary step, three authors (OG, AE, and CO) identified general areas of interest to inform the design of two survey instruments. The primary goal of one survey instrument was to capture attitudes toward adopting genome-guided prescribing through CDS prior to enlisting in the CLIPMERGE PGx study. The primary goal for the second survey was to capture physician uptake of genome-guided prescribing through CDS and their opinions. One author (CO) drafted survey questions that also incorporated general areas of interest, question style, data analysis, and the existing literature.

For the survey administered prior to enlisting in the CLIPMERGE PGx study, three authors collectively agreed to adapt the Evidence Based Practice Attitude Scale (EBPAS) to this study, and to develop additional 2 to 5-point scale questions. We scaled the additional questions to four constructs: perceived usefulness (two items, e.g., “Please indicate your level of agreement with the following statement: patient genotype data is useful for making prescribing decisions.”), awareness (two items, e.g., “How aware are you of genome-guided prescribing?”), experience (two items, e.g., “How often do you use decision support aids?”), and comfort with technology (two items, e.g., “How comfortable are you with using computers?”). The adapted EBPAS is described in the following section. This survey was split into two short-form questionnaires that were administered before and after one-hour training sessions. Details about provider recruitment and survey procedures are described elsewhere [9]. Details about collected baseline quantitative measures using this survey are in the “Overview of measures” section.

For the physician uptake and opinion survey, the three authors agreed to develop 2 to 5-point scale questions. All questions were scaled to three constructs: use (three items, e.g., “Did you use the prescribing-related decision support message to inform your prescribing decision?”), perceived usefulness (three items, e.g., “How useful was the prescribing-related decision support message embedded in the patient electronic health record?”), and confidence in prescribing (four items, e.g., “How did confidence in your prescribing decision change in this case?”). This work analyzes data from the survey instrument used to capture physician attitudes prior to enlisting in the CLIPMERGE PGx project. The remainder of the manuscript therefore focuses on that survey instrument.

### 2.3. Adapting the Evidence Based Practice Attitude Scale

The Evidence Based Practice Attitude Scale (EBPAS) [17,18] measures attitudes toward adopting evidence-based practices. The scale was adapted to measure attitudes toward adopting genome-informed interventions (EBPAS-GII). The EBPAS-GII contains twelve items to measure attitudes toward adoption on three subscales that parallel the original EBPAS: (a) intuitive *Appeal* of genome-informed interventions; (b) *Openness* to new practices; and (c) perceived *Divergence* of usual practice with research-based/academically developed genome-informed interventions. Items are measured on a 5-point scale response format with anchors of “not at all” to “to a very great extent” (See Table 1 and Table 2). Openness and divergence subscale questions were included in the pre-training questionnaire. Appeal subscale questions were included in the post-training session questionnaire. Computing a total score for subscale items created the subscale scores. All items from the Divergence subscale were reverse scored. The total scale score was the sum of all subscale scores.

### 2.4. Overview of Measures

Descriptions of the quantitative measures for this study are shown in Table 3. The socially desirable response (SDR) scale was one possible covariate.

**Table 1:** Pre-training session adapted evidence-based practice attitude scale items and scoring.

Item	Subscale	Question
*Pre-training session questionnaire*		*Please indicate the extent to which you agree with each item.* *0-not at all|1-to a slight extent|2-to a moderate extent|3-to a great extent|4-to a very great extent*
1	Openness	I like to use new types of therapy/interventions to help my patients.
2	Openness	I am willing to try genome-guided prescribing even if I have to use a decision support aid.
3	Divergence	I know better than academic researchers how to care for my patients.
4	Openness	I am willing to use new and different types of genome-guided prescribing decision support aids developed by researchers.
5	Divergence	Research based genome-guided prescribing decision support aids are not clinically useful.
6	Divergence	Clinical experience is more important than using decision support aids.
7	Divergence	I would not use genome-guided prescribing decision support aids.
8	Openness	I would try a new decision support aid even if it were very different from what I am used to doing.

**Table 2:** Post-training session adapted evidence-based practice attitude scale items and scoring.

Item	Subscale	Question
*Post-training session questionnaire*		*How likely would you be to adopt a genome-guided prescribing decision support aid if:* *0-not at all|1-to a slight extent|2-to a moderate extent|3-to a great extent|4-to a very great extent*
1	Appeal	It was intuitively appealing?
2	Appeal	It “made sense” to you?
3	Appeal	It was being used by colleagues who were happy with it?
4	Appeal	You felt you had enough training to use it correctly?

### 2.5. Preliminary Assessment of Survey Instrument Content and Face Validity

We assessed the content and face validity of our survey instrument with a panel of individuals with clinical, genomics, and informatics expertise (*n* = 7). The panel assisted with determining the appropriate number of instrument questions and appropriate language to use given the target population. In addition, the panel decided to ask questions not related to the constructs. These included additional questions about attitudes toward personal genome testing (PGT), general questions about demographics (e.g., years practicing), and open-ended questions to encourage exploratory research. We later included the 5-item socially desirable response scale (SDRS-5) [19] to facilitate adjusting for socially desirable responses.

### 2.6. Data Analyses

We assessed the internal consistency reliability of EBPAS-GII (total and subscales) and SDRS-5 by calculating Cronbach’s coefficient alpha [20]. Scales with reliabilities of 0.70 or greater are recommended for group comparisons. We report frequencies of physician characteristics (awareness, experience, and perceived usefulness), attitudes about PGT services, and comfort using technology. We also collapsed predictor variables into binary values and conducted independent *t*-test analyses to determine if there were differences in attitudes about genome-guided prescribing through CDS (*i.e*., EBPAS-GII summary score) based on physician characteristics, attitudes about PGT services, and comfort using technology. Multivariable linear regressions were run to test whether associations existed after controlling for socially desirable responses. We report *p*-values, adjusted *p*-values, coefficient of determination (*R*^2^), as well as mean EBPAS-GII summary scores. All analyses were conducted in Stata 11.2. For all tests, we report *p*-values with an alpha level of 0.05.

**Table 3:** Provider baseline measures.

Concept	Definition	Items ^1^
Attitudes toward adoption	Attitudes toward adoption of genome-guided prescribing CDS	Twelve items (Evidence Based Practice Attitude Scale—Genome Informed Interventions, EBPAS-GII) ^2^
Perceived usefulness	Perceived usefulness of genome-guided prescribing CDS	Two items: patient genotype data is useful for making prescribing decisions; decision support aids are useful for making prescribing decisions Rating scales range from 1 to 5: 1 = definitely false 2 = mostly false 3 = don’t know 4 = mostly true 5 = definitely true
Awareness	Awareness of genome-guided prescribing CDS	Two items: how aware are you of decision support aids; how aware are you of genome-guided prescribing Rating scales are binary: 0 = unaware of use 1 = aware of use
Experience	Experience with genome-guided prescribing CDS	Two items: how often do you use decision support aids; how often do you perform genome-guided prescribing Rating scales range from 0 to 2: 0 = never use 1 = sometimes use 2 = often use
Comfort using technology	Comfort using computers and the local electronic health record system	Two items: how comfortable are you with using computers; how comfortable are you with using Epic (the local electronic health record system)? Rating scale ranges from 1 to 5: 1 = not at all comfortable 2 = not very comfortable 3 = neither comfortable or uncomfortable 4 = comfortable 5 = very comfortable
Covariate		Socially desirable response (SDR) scale ^3^

^1^ Items from baseline questionnaires distributed during the training session. ^2^ Adapted from the Evidence Based Practice Attitude Scale [17,18] (See Table 1 and Table 2). ^3^ Socially desirable response scale (SDRS-5) [19].

## 3. Results

From early August 2012 to late June 2013 the survey was administered during seven one-hour teaching sessions. 104 Internal Medicine physicians from ISMMS completed the survey, and 101 were enlisted into the overall study. The data of two individuals who completed the survey but did not consent to participate in the study were removed from our analyses. The demographics of the study participants are reported in Table 4. Eighty-eight percent of physicians were residency program trainees (PGY1 to PGY4).

**Table 4:** Icahn School of Medicine at Mount Sinai (ISMMS) internal medicine physician demographics.

Characteristics	General Internal Medicine Physicians (*N* = 101) *N* (%)
Gender	
Male	40 (39.6)
Female	61 (60.4)
Years practicing	
PGY1	75 (75.0)
PGY2	9 (9.0)
PGY3	3 (3.0)
PGY4	2 (2.0)
Fellow	1 (1.0)
Attending	10 (10.0)

Table 5 presents means and SDs of the EBPAS-GII and SDRS-5 scores for survey respondents. Internal consistency reliability alpha coefficients are presented for three EBPAS-GII subscales: appeal, openness, and divergence. The number of respondents for each subscale was less than 101 due to missing data. The divergence subscale cannot be used as an independent indicator (Cronbach’s alpha < 0.7), therefore subsequent analyses were performed with EBPAS-GII summary scores (i.e., overall attitudes toward adopting genome-guided prescribing CDS).

**Table 5:** EBPAS-GII and SDRS-5 scores.

Scale/Subscale	# of items	N	Mean	SD	Range	Alpha
EBPAS-GII Total Score	12	91	36.81	5.89	25–51	0.78 ^a^
Appeal	4	96	11.45	2.76	4–16	0.82
Openness	4	98	10.58	2.65	6–16	0.81
Divergence	4	99	12.02	2.47	1–16	0.58
SDRS-5 Total Score	5	100	0.98	1.23	0–5	0.73 ^a^

^a^ Published psychometric properties of EBPAS total (15-items, alpha = 0.79 [18]) and SDRS-5 (alpha = 0.68 [19]).

Table 6 presents EBPAS-GII total scale scores by physician characteristics, attitudes about PGT services, and comfort using technology. We measured physician characteristics including awareness of, experience with, and perceived usefulness of decision support aids and genome-guided prescribing. Sample sizes for regression models varied slightly because of missing responses.

**Table 6:** EBPAS-GII total scale scores by predictor variables. NS: not statistically significant.

Predictor Variable	EBPAS-GII (Total)					
	*N*	*Mean*	*SD*	*p*	*p_adj_*	*R*^2^
**Awareness**						
How aware are you of decision support aids?				NS	NS	0.02
unaware of use	16	36.56	5.77			
aware of use	75	36.87	5.96			
How aware are you of genome-guided prescribing?				NS	NS	0.02
unaware of use	56	36.75	5.25			
aware of use	35	36.91	6.88			
**Experience**						
How often do you use decision support aids?				NS	NS	0.02
never use	8	37.38	5.95			
sometimes use/often use	82	36.78	5.95			
How often do you perform genome-guided prescribing?				NS	NS	0.02
never use	83	36.90	5.89			
sometimes use/often use	8	35.88	6.29			
**Usefulness**						
Decision support aids are useful for making prescribing decisions?				NS	NS	0.02
definitely false/mostly false/don’t know	18	35.94	5.74			
mostly true/definitely true	73	37.03	5.95			
Patient genotype data is useful for making prescribing decisions?				0.004	0.004	0.11
definitely false/mostly false/don’t know	44	35.02	5.10			
mostly true/definitely true	47	38.49	6.14			
**PGT companies**						
Have you heard of PGT companies?				0.045	NS	0.07
unaware of PGT	50	35.74	6.00			
aware of PGT	40	38.25	5.56			
Have you used/would you consider using PGT services?				NS	NS	0.03
would not use	22	36.09	5.11			
would use/did use	65	36.69	6.11			
I know enough about genetics and genomics to understand PGT test results?				NS	NS	0.04
definitely false/mostly false/don’t know	36	35.58	5.88			
mostly true/definitely true	54	37.50	5.80			
**Comfort with Technology**						
How comfortable are you with using computers?				NS	NS	0.06
neither comfortable or uncomfortable/not comfortable	23	34.91	6.19			
comfortable/very comfortable	68	37.46	5.69			
How comfortable are you with using Epic?				NS	NS	0.04
neither comfortable or uncomfortable/not comfortable	58	36.36	5.72			
comfortable/very comfortable	33	37.61	6.19			
**Years Practicing**						
PGY1 *vs*. PGY2+				NS	NS	0.02
PGY1	69	37.01	5.68			
PGY2+	21	36.67	6.37			

**Figure 1:**
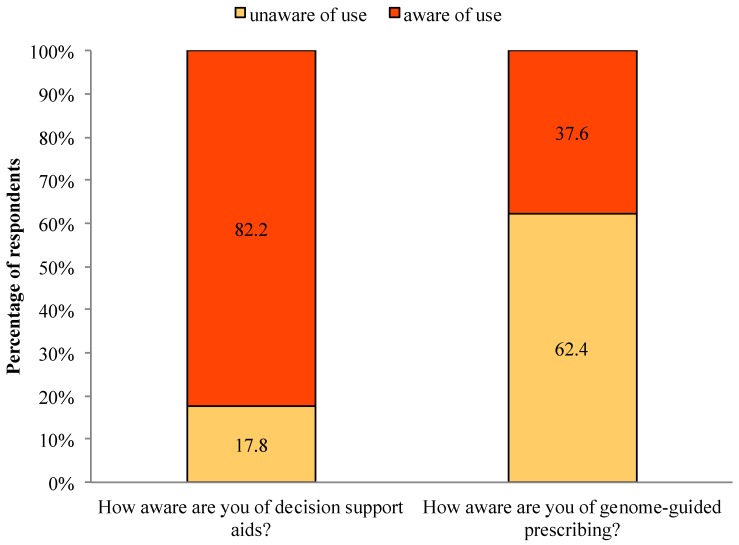
Awareness of decision support aids and genome-guided prescribing.

Among all respondents, 82.2% were aware of decision support aids and 17.8% were unaware. In contrast, 37.6% were aware of genome-guided prescribing, whereas 62.4% were unaware (See Figure 1). We found no significant associations between physician awareness of decision support aids or genome-guided prescribing and EBPAS-GII total score.

**Figure 2:**
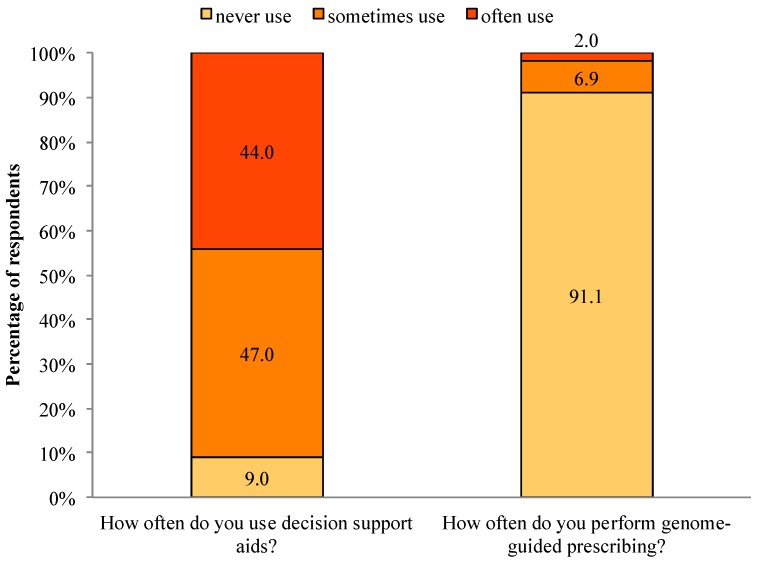
Experience with decision support aids and genome-guided prescribing.

The majority (91.0%) of respondents sometimes or often use decision support aids, with only 9.0% never using decision support aids. In contrast, only 8.9% of respondents sometimes or often use genome-guided prescribing, compared to 91.1% who never use genome-guided prescribing (See Figure 2). We found no significant associations between physician experience with decision support aids or genome-guided prescribing and EBPAS-GII total score.

**Figure 3:**
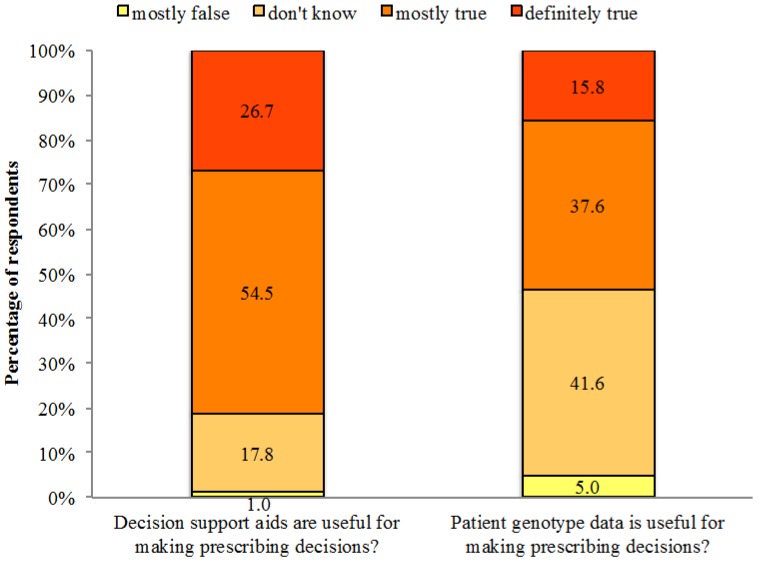
Usefulness of decision support aids and genotype data for making prescribing decisions.

Notably, 81.2% of respondents thought it was mostly true or definitely true that decision support aids are useful for making prescribing decisions, whereas 18.8% did not know or thought it was mostly false that decision support aids are useful for making prescribing decisions. Regarding genetic information, 53.4% of respondents thought it was mostly true or definitely true that genotype data are useful for making prescribing decisions, compared to 46.6% who did not know or thought it was mostly false that genotype data are useful for making prescribing decisions (See Figure 3). We found no significant association between the perceived usefulness of decision support aids and EBPAS-GII total score. However, physicians who thought genotype data were useful for making prescribing decisions were more likely to have higher EBPAS-GII total scores after controlling for socially desirable responses (*p* = 0.004). This regression model with physician perceptions about the usefulness of genome-guided prescribing and SDRS-5 variables explained 11% of variation in EBPAS-GII total score.

In response to questions about PGT companies, 41% of respondents were aware of PGT companies, compared to 59% who were unaware. Although only 1% did currently use PGT services, 75% of respondents indicated that they would use PGT services, while 24% indicated they would not use PGT services. Regarding physician understanding of genetics, 58.6% of respondents thought it was mostly true or definitely true that they know enough about genetics and genomics to understand PGT test results (See Table 7). We found no significant association between measures of attitudes about PGT services and EBPAS-GII total score after controlling for socially desirable responses.

**Table 7:** Attitudes about personal genome testing companies.

Survey Question	*N* (%)
**Have you heard of PGT companies?**	
unaware of PGTs	59 (59.0%)
aware of PGTs	41 (41.0%)
**Have you used/would you consider using PGT services?**	
would not use	23 (24.0%)
would use	72 (75.0%)
did use	1 (1.0%)
**I know enough about genetics and genomics to understand PGT test results**	
definitely false	6 (6.1%)
mostly false	8 (8.1%)
don’t know	27 (27.3%)
mostly true	51 (51.5%)
definitely true	7 (7.1%)

**Figure 4:**
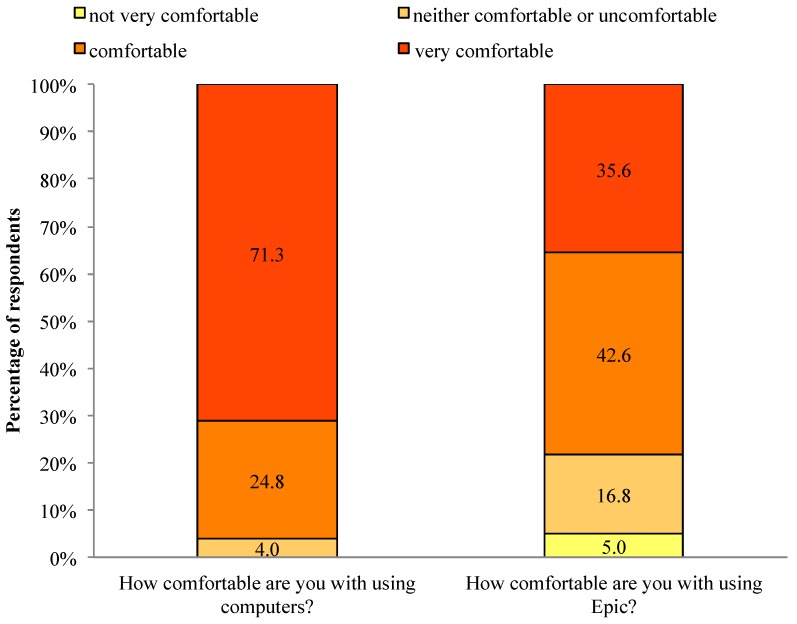
Comfort with technology.

In response to questions about physician comfort with technology, 96.1% of respondents were comfortable or very comfortable with computers, and 78.2% of respondents were comfortable or very comfortable with the local EHR system (See Figure 4). We found no significant association between comfort with computers or with using the local EHR system and EBPAS-GII total score.

Finally, in comparing PGY1 interns with more experienced (PGY2+) residents and physicians, we found no significant association between years practicing and EBPAS-GII total score.

## 4. Discussion

We developed a study instrument for assessing provider attitudes toward adoption of enome-guided prescribing through CDS that includes a modified Evidence Based Practice Attitude Scale (*i.e*., EBPAS-GII). We generated internal consistency measures for the modified scale and the Cronbach’s alpha was 0.78, which is comparable with the original scale (alpha = 0.79). This instrument also facilitated assessing physician characteristics (perceptions about the usefulness of, experience with, and awareness of genome-guided prescribing through CDS), attitudes about PGT services, and comfort using technology. When applied to our CLIPMERGE PGx physician cohort, physicians who perceived genotype data to be useful in making prescribing decisions had more positive attitudes toward adopting genome-guided prescribing through CDS.

In this pilot study, we surveyed 101 General Internal Medicine physicians from ISMMS. The majority were residency program trainees (~88%). Prior to enlisting into CLIPMERGE PGx most physicians were aware of and used decision support aids (82.2% and 91%, respectively). Few physicians, however, were aware of and used genome-guided prescribing (37.6% and 8.9%, respectively). The majority of physicians perceived decision support aids to be useful for making prescribing decisions (81.2%, answered mostly true or definitely true). The majority of physicians also perceived genotype data to be useful for making prescribing decisions (53.4%, answered mostly true or definitely true). Many physicians, however, were indifferent about the usefulness of decision support aids and genotype data for making prescribing decisions (18.8% do not know if decision support aids are useful and 46.6% do not know if genotype data are useful). When assessing physicians opinions about PGT companies, most physicians had not heard of PGT companies (59%), but were willing to use PGT services (75%). Only one respondent had used PGT services him/herself. In addition, most physicians believed they knew enough about genetics and genomics to understand PGT test results (58.6%, answered mostly true or definitely true). When assessing comfort with technology, the majority of physicians were comfortable or very comfortable with computers and with using the local EHR system specifically (96.1% and 78.2%, respectively). Finally, results indicated that physician attitude toward adopting genome-guided CDS varied by perceived usefulness of genotype data in making prescribing decisions. All other associations were non-significant and thus may be distinct constructs worthy of assessing in future work. Another study, for example, is underway to assess the impact of measurements from this study on the uptake of genome-guided CDS and physician opinions about genome-guided CDS.

This study highlights that internal medicine physicians have a deficit in their familiarity and comfort interpreting and using genomic information. These are significant barriers that are echoed in other studies [3,4,5] and that will likely influence widespread implementation of genomic medicine over the coming years. These barriers are especially relevant to internal medicine, where the use of genomic information is likely to be more generally applied to the overall management of patients, both in terms of response to medications (pharmacogenomics) and in terms of complex chronic disease risk. In comparison to some specialties such as oncology, internal medicine trainees have less exposure to the utility of genomic information. Our hope is to develop best practices through implementation research programs, such as CLIPMERGE PGx, that will enable the appropriate use of genomic information in clinical care and help to unburden providers from the arduous task of remaining current in a rapidly changing field, across the spectrum of clinical specialties.

### Limitations and Strengths

ISMMS was early to prioritize widespread genomic medicine implementation and as such, probably does not fairly reflect the state of affairs at medical schools and healthcare institutions across the U.S. This study would benefit from additional assessment of our survey instrument in other settings and construct validity analyses to support the broad use of survey instrument measures. It would be beneficial, for instance, to assess the convergent validity of the EBPAS-GII measures by determining how correlated they are with EBPAS measures. Even so, we believe that our assessment of the face and content validity of our survey instrument was sufficient for this pilot study. In addition, the EBPAS-GII survey items were derived from a valid and reliable survey instrument.

This study also focused primarily on internal medicine trainees, who function as primary care physicians and are therefore more likely to be confronted with treatment-relevant genomic information than physicians in other specialties. It is possible that the views of this cohort do not accurately reflect or capture the views of non-trainees, or those of trainees or physicians in other specialties. As the CLIPMERGE PGx program expands, we plan to include physicians from all specialties in our research and will be analyzing further how types of experience, as well as years of experience, may shape physician views. It is hoped that lessons we learn may be disseminated to other institutions embarking on similar programs in the future and may help shape genomic medicine implementation and education efforts in general.

## 5. Conclusions

The results of this study have already informed us that in order to increase adoption, genome-guided CDS must explicitly highlight why the information we provide is useful in helping physicians make prescribing decisions. This has reinforced the importance of getting regular feedback and guidance from our enrolled physicians when designing genome-guided CDS. In addition, given awareness of genome-guided prescribing among internal medicine physicians is low, we must prioritize the genomics education of medical students, trainees and physicians from all disciplines. Genomics now forms a component of the medical school curriculum at ISMMS, and our group and others are providing genomic medicine educational sessions for providers across the Mount Sinai Health System.
